# Optimizing Antimony Speciation Analysis via Frontal Chromatography–ICP-MS to Explore the Release of PET Additives

**DOI:** 10.3390/molecules29122870

**Published:** 2024-06-16

**Authors:** Alejandro R. López, Gilberto Binda, Gianluca Roncoroni, Sandro Recchia, Damiano Monticelli, Davide Spanu

**Affiliations:** 1 University School for Advanced Studies IUSS Pavia, 27100 Pavia, Italy; alejandro.ruiz@iusspavia.it; 2 Department of Science and High Technology, University of Insubria, Via Valleggio 11, 22100 Como, Italy; gilberto.binda@niva.no (G.B.); groncoroni1@uninsubria.it (G.R.); sandro.recchia@uninsubria.it (S.R.); 3 Norwegian Institute for Water Research (NIVA), Økernveien 94, 0579 Oslo, Norway

**Keywords:** antimony, speciation analysis, plastic pollution, environmental monitoring, analytical chemistry, polyethylene terephthalate, green analytical chemistry, chromatography, ICP-MS, plastic additives

## Abstract

Antimony (Sb) contamination poses significant environmental and health concerns due to its toxic nature and widespread presence, largely from anthropogenic activities. This study addresses the urgent need for an accurate speciation analysis of Sb, particularly in water sources, emphasizing its migration from polyethylene terephthalate (PET) plastic materials. Current methodologies primarily focus on total Sb content, leaving a critical knowledge gap for its speciation. Here, we present a novel analytical approach utilizing frontal chromatography coupled with inductively coupled plasma mass spectrometry (FC-ICP-MS) for the rapid speciation analysis of Sb(III) and Sb(V) in water. Systematic optimization of the FC-ICP-MS method was achieved through multivariate data analysis, resulting in a remarkably short analysis time of 150 s with a limit of detection below 1 ng kg^−1^. The optimized method was then applied to characterize PET leaching, revealing a marked effect of the plastic aging and manufacturing process not only on the total amount of Sb released but also on the nature of leached Sb species. This evidence demonstrates the effectiveness of the FC-ICP-MS approach in addressing such an environmental concern, benchmarking a new standard for Sb speciation analysis in consideration of its simplicity, cost effectiveness, greenness, and broad applicability in environmental and health monitoring.

## 1. Introduction

Antimony (Sb) stands out as a significant environmental concern due to its toxic nature and widespread presence across various environmental compartments [1,2]. Even though its natural presence in uncontaminated waters is limited (below 1 μg/L) [3], anthropogenic activities such as mining, non-ferrous ore processing, and plastic production can lead to abundant antimony release into the environment, impacting water sources [4,5,6].

Drinking water contaminated with Sb poses potential health risks, ranging from short-term effects like nausea and diarrhea to long-term carcinogenic effects associated with chronic exposure [7]. Regulatory organizations worldwide have established permissible concentrations for total Sb in drinking water (e.g., 10 μg/L in Europe [8]), yet specific regulations for Sb(III) and Sb(V) species are lacking, despite Sb(III) being recognized as ten times more toxic than Sb(V) [1,9]. Also, Sb(III) has been categorized as likely carcinogenic to humans by the International Agency for Research on Cancer of the World Health Organization, while Sb(V) cannot be classified in regard to its carcinogenicity [10,11].

Particularly concerning is the migration of Sb species in mineral water from polyethylene terephthalate (PET) materials, such as drinking water bottles [12,13,14]. PET production utilizes antimony trioxide (Sb_2_O_3_) as a catalyst in the transesterification and polycondensation stages during manufacturing, leading to the entrapment of Sb within the polymer matrix post-production, with concentrations reaching up to 0.07% wt. [14]. This characteristic raises concerns regarding the potential adverse effects of Sb species leached from PET bottles into water and beverages, necessitating a thorough evaluation of this phenomenon. However, the current literature predominantly focuses on the total migrated amount of Sb [12,13,15,16,17], with a very poor number of works focusing on its speciation [18,19], thus leaving the recognition of different Sb species as a significant knowledge gap. Moreover, the diverse PET production technologies lead to an extreme variability in the total amount of Sb present in the polymer and, importantly, Sb speciation (likely due to partial oxidation of Sb(III) during manufacturing) [20,21]. This evidence complicates the modelling of the migration process, underscoring the need for an in-depth investigation into Sb speciation in water. Beyond a human health risk assessment, such a study is crucial for understanding the potential environmental implications of Sb released from improperly managed and dispersed PET plastic materials. Various environmental factors, including temperature, light exposure, and biological degradation, may in fact contribute significantly to the release of additives into the aquatic environment [22,23,24]. Thus, there is an urgent need to investigate the speciation of Sb migrated from PET polymers in water to gain new insights into Sb migration mechanisms.

The development of accurate analytical methods for Sb speciation at ultra-trace levels can surely help in approaching this goal. Over the past decades, efforts have been dedicated to advancing analytical methodologies in this field (see the yearly updated review on advances in elemental speciation methods [25], and additional references [26,27,28,29,30,31,32,33,34,35]). For instance, in recent years, many non-chromatographic protocols, encompassing electroanalytical [33,34] and spectrometric techniques [35], have been developed. However, their implementation necessitates various sample pretreatment steps, including pre-reduction [26], solid-phase extraction [36], solvent extraction [37], or cloud-point extraction [38]. These additional procedures raise doubts regarding the feasibility of employing non-chromatographic approaches for routine analysis. In contrast, chromatographic methods typically offer shorter analysis times and higher productivity. However, they often require extensive sample preparation too. These include Sb species complexation [39], the removal of particulate matter through pre-digestion [40], and the preconcentration of Sb species [40]. A widely adopted and effective sample treatment is hydride generation [25,26], which offers advantages in terms of sensitivity and selectivity for Sb speciation. Despite these benefits, there are challenges from a green analytical chemistry perspective due to the reliance on toxic chemical reagents, waste generation, energy consumption, and the complexity of the instrumentation.

Therefore, efforts to develop new greener procedures characterized by an improved throughput, simplified (or absent) sample pretreatments, reduced costs (e.g., minimizing the requirement for expensive separation systems and limiting reagent consumption), and compatibility with highly sensitive analytical techniques such as ICP-MS (for example, utilizing a HNO_3_ eluent, as demonstrated only in a limited number of studies) would significantly foster the monitoring of Sb ultra-traces and the evaluation of risks related to these chemicals. In response to these needs, our research group has undertaken the preliminary development of an analytical method based on frontal chromatography (FC) coupled with ICP-MS (FC-ICP-MS) for Sb speciation [41]. This technique, using a short low-pressure column with a limited resolution fed by a peristaltic pump, is strongly indicated when the high resolution of HPLC is superfluous, i.e., when two species have a markedly different nature (e.g., ionic/non-ionic) [42,43,44,45], as the case of Sb(III)/Sb(V) in an acid environment [41]. This approach offers a promising avenue for rapid speciation analysis of ultra-trace contaminants, as demonstrated by our work on the speciation of inorganic arsenic [42] and the selective determination of methylmercury [43,44] and hexavalent chromium [45].

Here, the innovative FC-ICP-MS method was systematically optimized by engineering the short column and varying key parameters such as the HNO_3_ concentration and sample flow rate. The method optimized by multivariate data elaboration allows us to accurately determine Sb(III) and Sb(V) in the outstandingly short time of 150 s, with a limit of detection lower than 1 ng kg^−1^. The optimized FC-ICP-MS method was finally tailored and applied to tackle the issue of the migration of Sb species from PET.

## 2. Results and Discussion

### 2.1. Analytical Method Optimization

The effective chromatographic separation of Sb(III) and Sb(V) species in a diluted HNO_3_ solution was achieved by employing the FC-ICP-MS setup with a strong cation-exchange resin as the stationary phase [41]. This separation is feasible with a low resolution and a simple FC system instead of a more complex and expensive HPLC one, as the two Sb species exhibit markedly distinct characteristics. As a matter of fact, at pH ≤ 1, Sb(III) exists as a cationic species (Sb(OH)_2_^+^), whereas Sb(V) is neutral (Sb(OH)_5_) [41].

Although FC-ICP-MS was already proved to provide reliable quantification of Sb(III) and Sb(V) ultra-traces in a proof-of-concept study [41], work must be conducted to systematically optimize the analytical methods by understanding the parameters controlling analysis time and resolution.

Here, optimum conditions were looked for by a design of experiments (DoE) which rigorously explores different (i) column lengths (25 and 50 mm), (ii) column widths (2 and 3 mm), (iii) sample flow rates (1.2, 1.45, and 1.7 mL min^−1^), and (iv) HNO_3_ concentrations (0.25, 0.5, and 0.75 M). The conditions employed for each experiment are summarized in Table 1 and Table S1.

**Figure 1:**
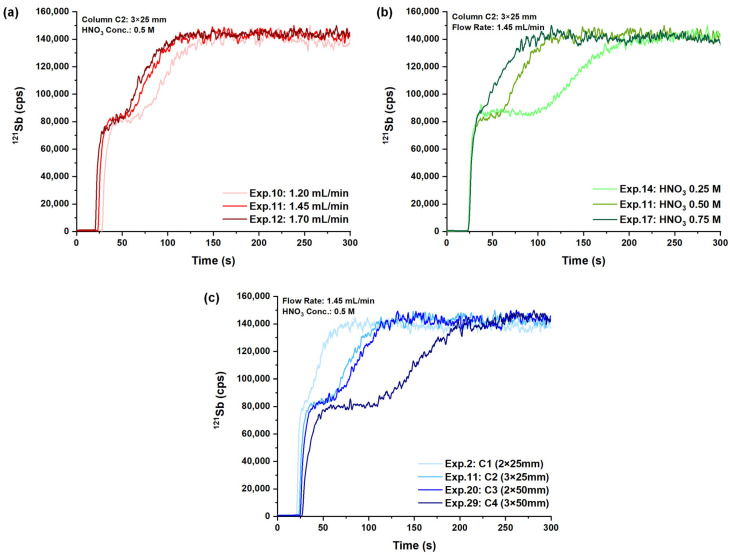
Effect of different chromatographic parameters: (**a**) sample flow rate; (**b**) nitric acid concentration; (**c**) column geometry. The chromatographic conditions for each test are reported in the corresponding panels.

The 36 resulting conditions (full-factorial DoE) were applied to the analysis of mixed Sb(III)-Sb(V) solutions (concentrations = 1 μg kg^−1^). The effect of each investigated parameter is depicted in Figure 1.

As a first observation, two distinct fronts are detected in most of the selected conditions: the first sigmoidal curve is related to the unretained species Sb(V), whereas the second one is ascribed to Sb(III) [41]. As Sb(V) does not interact with the cation-exchange resin, the shape of the Sb(V) signal is affected only by the sample flow rate and the column geometry (see overlapped Sb(V) profiles in Figure 1b at varying HNO_3_ concentrations). In more detail, increasing the sample flow rate shortens the analysis time, whereas reducing the column volume mainly reduces front sharpness even for unretained species. Differently, cationic Sb(III) interacts with the stationary phase, and all the chromatographic conditions affect the time necessary for its elution and the width of its front. Similarly to Sb(V), the increase in the sample flow rate and the decrease in the column size promote the fast elution of Sb(III). On the other hand, higher concentrations of HNO_3_ markedly improve the analysis time, as H^+^ species facilitate the fast elution of Sb(OH)_2_^+^.

**Figure 2:**
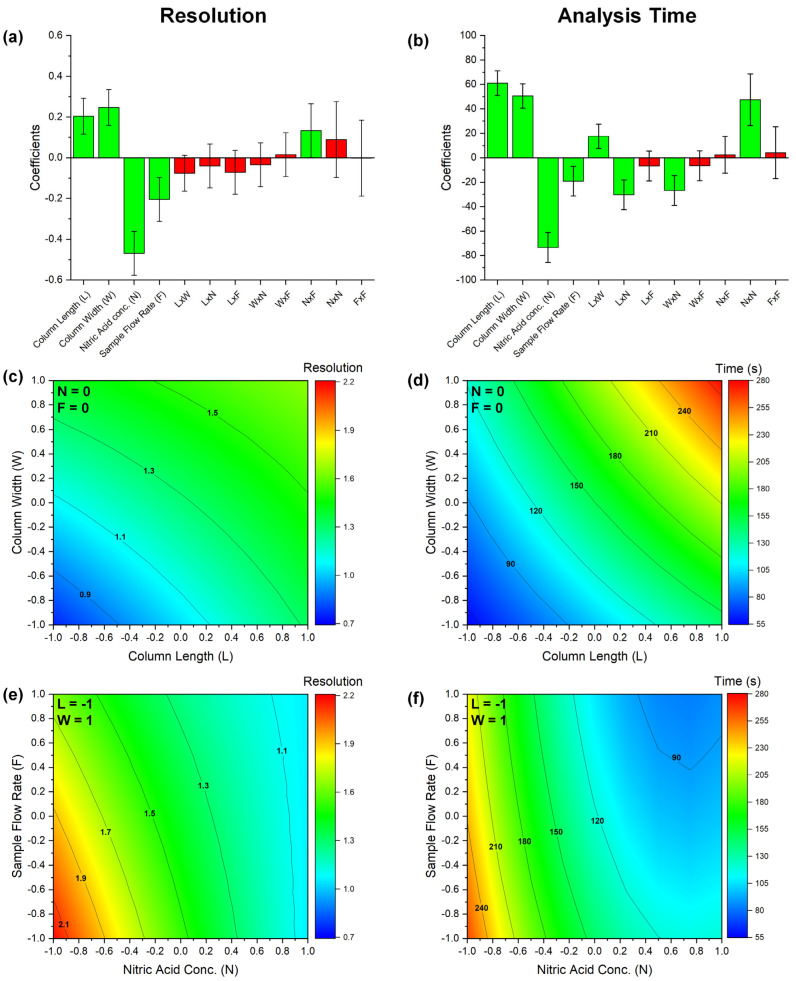
Results of multivariate regression analysis on the data presented in Table S1 to model chromatographic resolution (left column) and analysis time (right column). Panels (**a**,**b**) display regression coefficients, where green bars mean statistically significant parameters with a *p*-value of ≤0.05 and red bars mean not statistically significant factors. Panels (**c**–**f**) illustrate surface responses, depicting the effect of (**c**,**d**) column size and (**e**,**f**) sample flow rate and nitric acid concentration on the resolution and analysis time. Chromatographic conditions are detailed in their respective panels.

The DoE was used to quantitatively analyze the entire experimental space, as explained above (Table 1): data were used to define conditions of optimal resolution with minimum analysis time. In addition, the model pinpointed the most influential parameters, advancing the knowledge of FC-ICP-MS. Details on how analysis time and resolution were estimated are outlined in the Supporting Information (Figure S1). Both parameters were modelled by second-order equations including interaction and quadratic terms (Figure 2). The goodness of the regression model is evident considering the very high Explained Variance (81.4% for the resolution model and 93.5% for the analysis time model) and the low and randomly distributed residuals, as depicted in Figure S2. As shown in Figure 2a,b, all the main effects show a statistically significant effect (*p*-value ≤ 0.05) on both resolution and analysis time: this evidence is well in line with the data reported in Figure 1, considering that these parameters are mainly defined by the Sb(III) profile. In more detail, the concentration of HNO_3_ is the most important factor governing both the resolution and analysis time, whereas interaction terms instead are generally poorly significant, except for those involving the column geometry and acid concentration, which show a notable effect on the analysis time. Finally, the significance of the quadratic term of nitric acid concentration on the analysis time evidenced the non-linear correlations between these two parameters. These observations and data illustrated in Figure 2c,d clearly show the usual chromatographic trends, namely that column length significantly improved resolution at the expense of increased analysis time.

Actually, only the intermediate column size, i.e., columns C2 (3 × 25 mm) and C3 (2 × 50 mm), may provide sufficient resolution in a reasonable time. The simultaneous effect of HNO_3_ concentration and sample flow rate was then evaluated for this column (Figure 2e,f). Here again, the intermediate values for these factors enable a balance between analysis time and resolution: a faster chromatographic run can be attained with high levels of both acid concentration and sample flow rate, but inadequate resolution is obtained.

These results hold general significance for FC-ICP-MS separations. They suggest that eluent strength, stationary phase volume, and flow rate are the factors defining resolution and analysis time: accordingly, these parameters only may be optimized.

**Figure 3:**
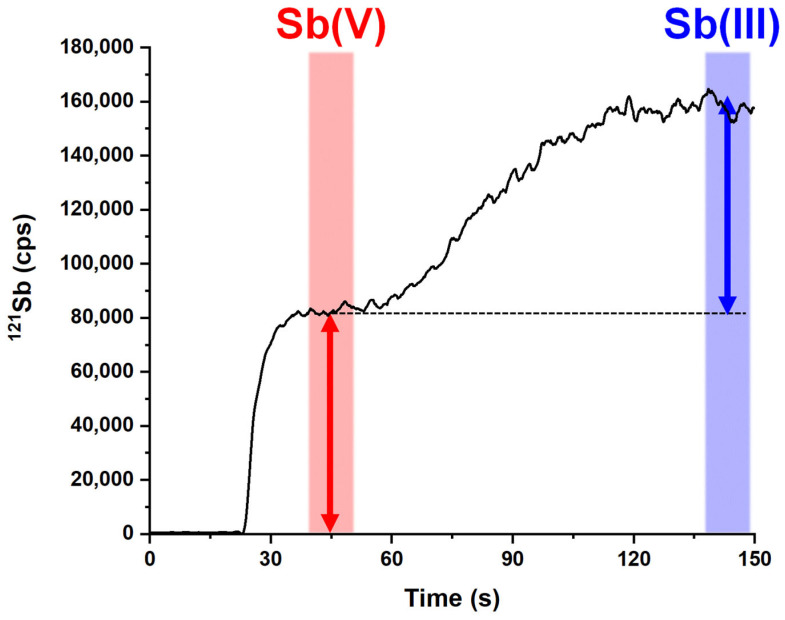
Frontal chromatogram obtained for an Sb(III)-Sb(V) mixture solution (both species at a concentration equal to 1 μg kg^−1^) under optimized conditions (column internal diameter = 3 mm, column length = 25 mm, HNO_3_ concentration = 0.5 M, and sample flow rate = 1.45 mL min^−1^, see condition 11 in Table S1). Red and blue regions denote the time intervals utilized to determine the height of the fronts (see red and blue arrows).

Summing up, we selected the optimal conditions as follows: column internal diameter = 3 mm, column length = 25 mm, HNO_3_ concentration = 0.5 M, and sample flow rate = 1.45 mL min^−1^ (condition 11 in Table S1). Working under these conditions, the time of analysis presented a value of 150 s, keeping a sufficient resolution (1.17). Thus, in addition to a strongly improved knowledge regarding FC separation, the optimization allowed to increase the analytical throughput by 20% compared to our previous work [41]. A frontal chromatogram of an Sb(V) and Sb(III) mixture obtained under the optimal conditions is depicted in Figure 3. The chromatogram features two well-defined signals that do not present a zone of Sb(III)-Sb(V) signal overlapping. This allows the reliable quantification of both species since the height of each front is proportional to the concentration of the corresponding species [42]. In more detail, after baseline subtraction, Sb(V) and Sb(III) concentrations were determined in the time ranges of 43–53 s and 140–150 s, respectively.

### 2.2. Analytical Performances of the Optimized Method

**Figure 4:**
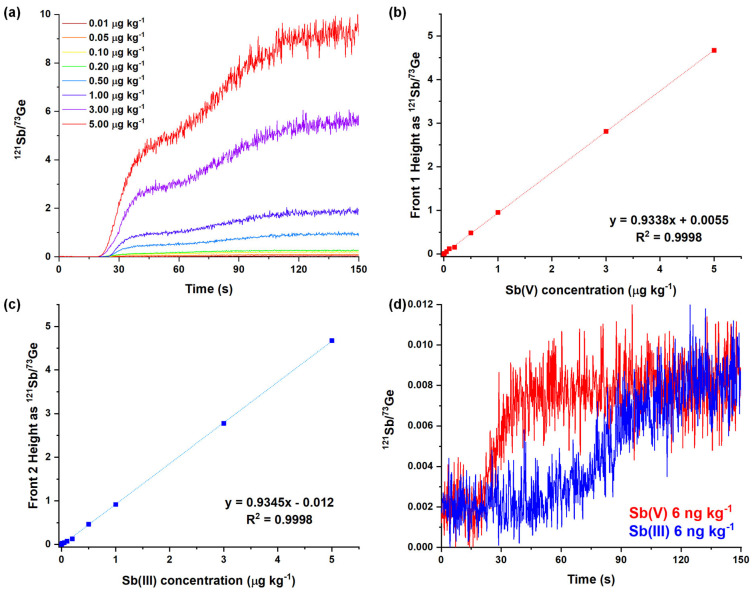
(**a**) Frontal chromatogram of equimolar Sb(III)-Sb(V) mixture solutions with varying concentrations: 0.01, 0.05, 0.1, 0.2, 0.5, 1, 3, and 5 μg kg^−1^. (**b**,**c**) Calibration lines obtained for Sb(V) and Sb(III). (**d**) Frontal chromatograms of 6 ng kg^−1^ Sb(III) and Sb(V) solutions analyzed for the estimation of LOD.

The linear range was examined in the interval between 0.01 and 5 μg kg^−1^ (Figure 4a). This was performed by directly analyzing Sb(III)-Sb(V) mixtures to save the time required for calibration and to verify the absence of interference among these species. A good linear correlation was found for both Sb species with R^2^ = 0.9998 (Figure 4b,c) and with similar sensitivities. Although higher Sb levels may fall within the linearity range, they were deliberately excluded, as they lie beyond the intended scope of application for this method.

The limits of detection (LODs) for both species were determined by analyzing ten replicates of 6 ng kg^−1^ standard solutions of pure Sb(III) and Sb(V). These Sb levels correspond to roughly five times the standard deviation of the background raw signal, following the guidance outlined in European standards for LOD and LOQ estimation [46]. The analysis revealed remarkably low LOD values of 0.7 and 0.5 ng kg^−1^ for Sb(V) and Sb(III), respectively, corresponding to a limit of quantification (LOQ) of 2.4 ng kg^−1^ for Sb(V) and 1.7 ng kg^−1^ in the case of Sb(III). Although these figures of merit were determined as suggested by international standards, we also decided to estimate the LOD of Sb(III) (the second eluted species) using analogous experiments in the presence of significant concentrations of Sb(V), as this condition may represent real samples. This involved performing ten replicate analyses of an Sb(III) 6 ng kg^−1^ solution containing 10, 100, and 500 ng kg^−1^ of Sb(V). Detection limits of 3.8, 6.4, and 5.2 ng kg^−1^ were found, respectively, highlighting a very limited effect of the occurrence of Sb(V) on the LOD for Sb(III).

The comparison of the figures of merit of the proposed FC-ICP-MS strategy to those of other chromatographic (HPLC) methods highlights one of the shortest analysis times (5 min including washout time) by the FC method, which also offers the widest linearity range as well as one of the lowest LOD values (refer to [41] and references therein for the sake of comparison): this establishes the developed method as a new standard in Sb speciation analysis. Additionally, the simplicity of the instrumental setup, evident in the absence of an HPLC system, coupled with the streamlined analytical protocol, outshines many existing methods. For instance, techniques relying on hydride generation (HG) necessitate specialized equipment and the continuous use of hazardous reagents like NaBH_4_, all without achieving comparable performance. Furthermore, even non-chromatographic methods involving preconcentration/extraction steps fail to deliver superior analytical results [36].

### 2.3. Application to PET Leaching Solutions

The optimized method was finally applied to the analysis of Sb species leached from PET plastic samples. Seven plastic samples were selected for this study: three differently colored environmental samples (blue, orange, and transparent), three differently colored virgin materials (blue, orange, and transparent), and one recycled PET sample (see additional details and sample label details in the Materials and Methods section). The polymer type was preliminary verified by an attenuated total reflectance infrared spectroscopy (ATR-IR) analysis (Figure S3) showing the characteristic peaks of C=O stretching (at 1715 cm^−1^), C-O stretching of aromatic esters (1245 cm^−1^), C-O stretching of aliphatic esters (1100 cm^−1^), and C-H bending (720 cm^−1^) [47].

This research focused on PET samples due to their well-documented elevated levels of Sb, a contaminant typically found in minimal quantities in other plastic materials. To validate this observation, we conducted an analysis of the total Sb species content in bulk PET using microwave-assisted acid digestion followed by ICP-MS determination. The results, summarized in Table 2, confirm this observation, with the total Sb content higher than 200 mg kg^−1^ for all the PET materials. Expectedly, a higher variability was observed in the environmental materials compared to the virgin ones, as environmental aging alters the polymer structure and homogeneity [24]. Notably, all the blue plastics exhibited the highest levels of total Sb, regardless of their origin (both environmental and virgin).

**Figure 5:**
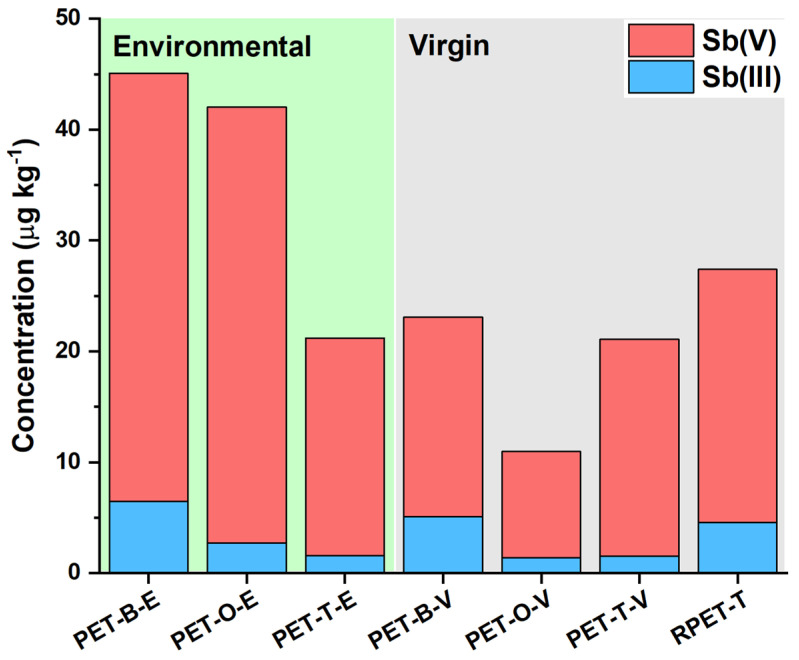
Concentration of Sb(III) and Sb(V) (μg kg^−1^ in solid sample) leached from PET samples after the ultrasound-assisted extraction in HNO_3_ 0.5 M.

The speciation data for the leaching of Sb species from the seven PET samples after ultrasound-assisted extraction in HNO_3_ 0.5 M are presented in Table 2 and illustrated in Figure 5. The environmental relevance of the selected extractant is significant, as analogous conditions are widely used for studying the leaching of physiosorbed and chemisorbed (or generally exchangeable) species in various solid environmental matrices. As a matter of fact, this approach is also employed in official standard methods (see, e.g., [48]), underscoring the relevance of the extraction protocol used [49,50]. The analytical protocol was validated by spiking an extraction solution with an Sb(III)-Sb(V) mixture (1 μg kg^−1^ for each species) and estimating the recovery of the added species after the 10 min long extraction. Recoveries of 95 ± 5% for Sb(V) and 93 ± 9% for Sb(III) were found, demonstrating the accuracy and suitability of the analytical protocol. These results also highlight the absence of Sb species loss and interconversion during the ultrasound-assisted extraction.

Data collected for PET samples show that environmental samples generally leach more Sb species compared to virgin and recycled samples, but the enhanced leaching is not caused by modification of the bulk properties. Actually, the aging process does not significantly affect the total Sb content in PET plastics, whereas it increases its leaching. The latter may be ascribed to a higher surface area and lower hydrophobicity of environmental samples, favoring water–polymer interactions, as observed in other studies with metallic additives [47,51]. Among the environmental samples, blue PET (PET-B-E) leaches the most total Sb, followed by orange (PET-O-E) and transparent (PET-T-E) PET. Recycled PET (RPET-T) shows higher leaching than virgin samples but lower than environmental samples, indicating a possible effect of the recycling process on the ability of PET to release Sb species. Regarding the speciation of Sb, Sb(V) is consistently higher in leachates compared to Sb(III) across all samples (Sb(V) fractions in the range of 78–94%), regardless of the origin of the plastic samples. Significant variations in leached Sb species concentration and distribution are instead found among different colors, suggesting that additives or pigments in colored PET might influence the chemistry of the leaching process, as well as the speciation of Sb during their production.

Summing up, the high variability in terms of leached Sb species is certainly related to both the different manufacturing processes and the degree of aging. These observations can help in understanding the environmental and health implications of Sb in PET products, guiding better recycling practices and material selection for reducing Sb exposure.

### 2.4. Evaluation of the Greenness of the Analytical Protocol

**Figure 6:**
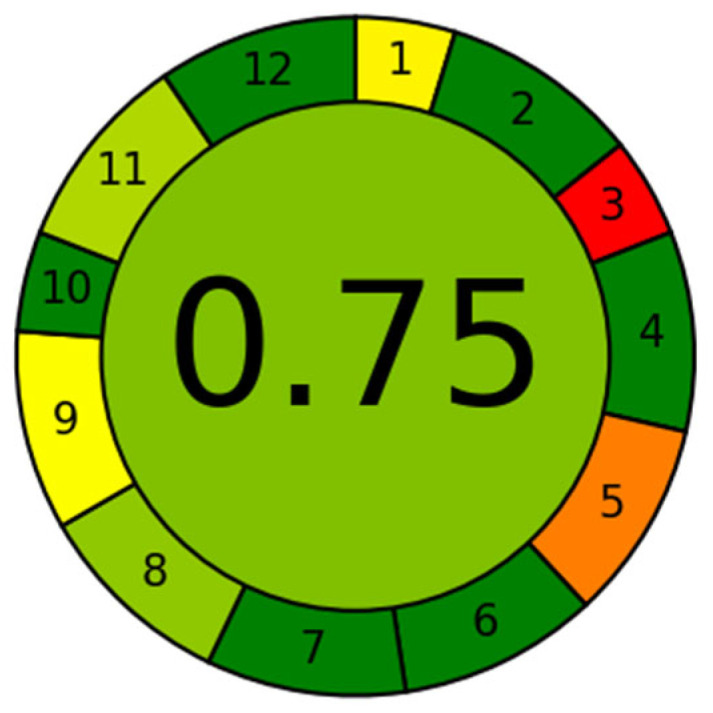
Results of AGREE analysis for the proposed analytical method. Lower weights were assigned to less relevant criteria: criteria 1 and 3 due to the unavailability of direct and in situ methods for tracing Sb species leached from solid materials, and criteria 10 as no organic solvents from biomasses are used.

Under the analytical chemistry point of view, the perfect integration of sample treatment with the analytical technique contributes significantly to fulfilling the 12 principles of green analytical chemistry [52]. As depicted in Figure 6, a commendable score of 0.75 out of 1.00 was achieved, based on the Analytical GREEnness metric approach (AGREE) [53]. This accomplishment is attributed to several factors, including the minimal sample requirement (criteria 2), streamlined procedural steps (criteria 4), the elimination of specific reagents such as derivatizing agents (criteria 6), the generation of limited analytical waste per sample (criteria 7), the good analytical throughput (criteria 8), and no specific issues related to the safety of the operator (criteria 12). Notably, the waste produced contains only a minimal volume of HNO_3_. The only drawbacks are its offline and ex situ nature (criteria 1 and 3), the lack of miniaturization (criteria 5), and the usage of a high-energy-consuming instrumentation like ICP-MS (criteria 9). However, these are common features shared by practically all other existing strategies for Sb speciation analysis.

To further highlight the advantages gained in the green analytical chemistry context with respect to existing methods, AGREE scores were determined for two HPLC-based representative protocols (see Figure S4): HPLC coupled to a hydride generation system connected to an atomic absorption spectrometer hydride generation (HPLC-HG-AAS) [28] and an HPLC-ICP-MS method [54] for water analysis. As depicted in Figure S4, both analytical protocols result less green compared to the one proposed in this work, with overall scores equal to 0.47 and 0.50, respectively. These much lower scores can be mostly ascribed to criteria 7, 10, and 11, which involve the usage of toxic reagents (e.g., NaBH_4_ and methanol necessary for HG and HPLC eluents), the abundant production of toxic wastes, and the usage of non-bio-based organic solvents.

## 3. Materials and Methods

### 3.1. Reagents

To prepare the stock standard solution of Sb(III) (1000 mg L^−1^), a proper amount of antimony(III) potassium tartrate hydrate (98% pure, Thermo Scientific Chemicals, Waltham, MA, USA) was dissolved in 30 mL of ultrapure water. The Antimony Standard for AAS (1000 mg L^−1^, Sigma-Aldrich, St. Louis, MO, USA, TracerCERT^®^) was used as stock standard solution of Sb(V). Intermediate standard solutions of different concentrations were prepared by diluting the concentrated solutions. In addition, a Ge standard solution (1000 mg L^−1^, Fluka, Everett, WA, USA, TraceCERT^®^) was employed as an internal standard by adding a proper volume of the Ge solution to each sample to reach a final concentration of 10 μg L^−1^. The choice of Ge as the internal standard is appropriate, as it is present as a neutral species (H_2_GeO_3_) in the employed analytical conditions [45], and thus it does not interact with the stationary phase.

Ultrapure nitric acid obtained by distillation from reagent-grade HNO_3_ (Carlo Erba, Milan, Italy, 65% pure) [55] was employed to acidify all the analyzed solutions.

All the samples were prepared by using ultrapure water produced by a Sartorius Arium mini UV Lab Water System (Varedo, Italy) and low-density polyethylene bottles after a decontamination procedure as follows: (I) soaking in a 0.4% *w*/*w* detergent solution (Nalgene L900, Thermo Scientific, Waltham, MA, USA) for a week; (II) soaking in a HNO_3_ solution (2% *w*/*w*) for a week; (III) soaking in a second HNO_3_ solution (2% *w*/*w*) for one week [56]. Bottles were thoroughly rinsed with ultrapure water between each step and before use.

### 3.2. Instrumental Setup and Analytical Procedure

Antimony detection was conducted using a Thermo Scientific ICAP Q inductively coupled plasma mass spectrometer (ICP-MS). Under optimized conditions, the separation of Sb species was achieved by employing a custom-made Polyether ether ketone (PEEK) column (internal diameter 3 mm, length 25 mm) packed with a strong cationic exchange resin (Dowex^®^ 50WX8 hydrogen form 200–400 mesh, Sigma-Aldrich). The packing media is retained within the column thanks to two HPLC frits. The column was positioned between the peristaltic pump (utilized for sample uptake) and the nebulizer of the ICP-MS and featured relatively large resin particles (55 ± 20 μm) and a short length: this permits minimal overpressure, thereby eliminating the need for an HPLC pump. Under optimal conditions, all the samples were acidified with ultrapure HNO_3_ reaching a final concentration of 0.5 M and introduced into the ICP-MS employing a sample flow rate equal to 1.45 mL min^−1^. During all ICP-MS analyses, the signals of ^121^Sb and ^73^Ge isotopes were monitored, with Germanium serving as the internal standard signal. Additional operating ICP-MS conditions are outlined in Table S2. The analysis was automated utilizing an autosampler (CETAC ASX-260 Autosampler, Thermo Scientific, Waltham, MA, USA), and the standard rinsing/cleaning procedure typically performed for automated ICP-MS determination was used to washout the line and the column. This means that after each sample analysis, the uptake tube is transferred to an external washout reservoir (HNO_3_ 0.5 M), which is then fed through the column thanks to the peristaltic pump. The washout process takes the same time as the sample analysis.

Concerning data elaboration, analysis time and resolution were estimated by calculating the first derivative curve and modelling each peak with a Gaussian function (refer to equation in Figure S1b). These parameters were finally calculated by applying the equations reported in Figure S1b, which adheres to the standard definitions in elution chromatography. Origin 2018 software (version 9.5.1.195, OriginLab, Northampton, MA, USA) was used for these calculations. Multivariate data analyses involved in DoE model computation were performed using CAT (Chemometric Agile Tool) software (freely downloadable from http://gruppochemiometria.it/index.php/software, 16 April 2024 version).

### 3.3. Sb(III) and Sb(V) Analysis in Plastic Samples

Seven plastic samples made of polyethylene terephthalate (PET) were analyzed in this work. The features of the samples are summarized in Table 3.

Environmental plastics were collected on a beach shore of Como Lake (45°58′37.9″ N, 9°15′03.8″ E), an oligo-mesotrophic lake in Northern Italy characterized by local littering and the longitudinal transport of plastic litter [57]. Figure S5 shows pictures of collected environmental samples. Virgin plastics were purchased as water and soft-drink bottles. Prior to any analysis on these samples, they were preliminary rinsed several times with ultrapure water to remove any deposited material.

Attenuated total reflectance infrared spectroscopy (ATR-IR) was used on all plastic samples to confirm their polymer composition. The analyses were carried out utilizing the Thermo Scientific™ Nicolet™ iS™ 10 FTIR Spectrometer based in the USA. Each sample underwent thirty-two scans, yielding relative standard deviations below 0.1%.

The leaching of Sb(III) and Sb(V) species was carried out by an ultrasound-assisted extraction lasting 10 min in HNO_3_ 0.5 M. An ultrasonic bath (Bandelin Sonorex Super RK 510 H, Berlin, Germany) filled with de-ionized water was used for this purpose. The temperature of the water bath, as monitored with an external thermocouple at the end of an extraction batch, never exceeded 25 °C. About 160 mg of sample (finely cut using ceramic scissors) was weighed and inserted into a low-density polyethylene (LDPE) bottle together with 10 g of the extractant (i.e., keeping the plastic–extractant mass ratio reported in the literature for the extraction of chemisorbed and physiosorbed species [49,50]). The solution, after the addition of Ge serving as the internal standard (a 10 μL addition) to correct any fluctuation in the Sb signal, was then directly analyzed by FC-ICP-MS. The analysis was performed three times.

### 3.4. Microwave-Assisted Acid Digestion of Plastic Samples for Total Sb Determination

The dissolution of plastic samples was performed through microwave-assisted acid digestion. An ETHOS One (Milestone MLS, Milan, Italy) MW digestion system equipped with 10 Polytetrafluoroethylene (PTFE) vessels was used for acid digestion. About 50 mg of the sample (finely cut using ceramic scissors) was weighed and inserted into a PTFE vessel. An amount of 5 mL of ultrapure HNO_3_, 0.2 mL of ultrapure H_2_SO_4_ (Analytika, Malden, MA, USA, 95% pure), and 1 mL of H_2_O_2_ (Fisher Chemical, Hampton, NH, USA, 30–32% for trace analysis) was added into the PTFE vessel. The samples were then digested by applying a temperature ramp reaching 200 °C. This temperature was kept for 1 h. The digested solution was then left to cool at room temperature. After mineralization, samples were transferred to low-density polyethylene (LDPE) bottles, diluted to 30 g with ultrapure water, and filtered with a 0.22 μm PTFE filter prior to the analysis via inductively coupled plasma-mass spectrometry (ICP-MS). Diluted solutions were analyzed using a Thermo Scientific ICAP Q ICP-MS using a He-collision cell in kinetic energy discrimination (KED) mode. Trace element quantification was performed by external calibration. Rhodium and Rhenium (sourced from a stock solution of 1000 mg L^−1^, Fluka, TraceCERT^®^) served as internal standards. The analysis was performed three times.

## 4. Conclusions

This study addresses the pressing need for the accurate speciation analysis of Sb in environmental matrices, with a particular focus on the migration of Sb species from PET plastic materials. The developed FC-ICP-MS method offers a rapid and sensitive approach for the speciation analysis of Sb(III) and Sb(V), with an optimized analysis time of 150 s and a low detection limit below 1 ng kg^−1^. These advantageous conditions were achieved after systematic experiments and a multivariate data analysis, examining all chromatographic effects involved in the Sb species separation. The eluent strength, flow rate, and column length emerged as the factors controlling resolution and analysis time, prompting these factors as the most relevant to be optimized during FC-ICPMS separations. This study establishes a new standard for Sb speciation analysis: it offers a streamlined, cost-effective, and environmentally friendly analytical approach (as proved by the green analytical chemistry metric AGREE) delivering excellent analytical performances. Thanks to its simplicity, the developed method holds promise for broad applicability in environmental monitoring, health risk assessment, and regulatory compliance.

In this context, the FC-ICP-MS approach was applied to the analysis of PET leaching solutions, revealing marked differences among the analyzed samples. Both the natural aging and the manufacturing process of PET, particularly the nature of the catalyst residues in the polymeric matrix, seem in fact to strongly influence the extent of Sb species leaching and their speciation. This highlights the potential environmental and health risks associated with PET materials. We anticipate that this study will facilitate the modelling of toxicological profiles, shedding light on the real hazards associated with PET bottles and Sb species. Moreover, it may prompt advocacy for stricter controls in PET production to mitigate Sb’s impact on ecosystems and human health. Finally, the environmental implications of Sb released from improperly mismanaged PET plastic may be explored in future research by investigating how factors like temperature, light exposure, and biological degradation contribute to Sb species leaching.

## Figures and Tables

**Table 1:** Chromatographic conditions (and their low, medium, and high levels used in DoE model computation for the sake of data standardization and centering) selected for all the experiments within the explored domain.

Level	Column Length (mm)	Column Width (mm)	HNO_3_ Concentration (M)	Sample Flow Rate (mL min^−1^)
−1	25	2	0.25	1.2
0	-	-	0.5	1.45
1	50	3	0.75	1.7

**Table 2:** Total Sb concentration (expressed as mg kg^−1^ in solid sample) resulting from microwave-assisted acid digestion of PET samples, alongside the concentration of Sb(III), Sb(V), and total Sb (μg kg^−1^ in solid sample) leached from PET samples after the ultrasound-assisted extraction in HNO_3_ 0.5 M. All the results are expressed as mean value ± one standard deviation (*n* = 3). Label legend: B = blue, O = orange, T = transparent, E = environmental plastic, V = virgin plastic (see Table 3 for additional details).

Sample	Total Sb Bulk Content	Leached Sb(V)	Leached Sb(III)	Leached Total Sb
PET-B-E	(311 ± 14) mg kg^−1^	(39 ± 5) μg kg^−1^	(6.5 ± 0.9) μg kg^−1^	(45 ± 5) μg kg^−1^
PET-O-E	(279 ± 46) mg kg^−1^	(39 ± 4) μg kg^−1^	(2.7 ± 0.3) μg kg^−1^	(42 ± 3) μg kg^−1^
PET-T-E	(263 ± 50) mg kg^−1^	(20 ± 1) μg kg^−1^	(1.6 ± 0.4) μg kg^−1^	(21 ± 1) μg kg^−1^
PET-B-V	(340 ± 18) mg kg^−1^	(18 ± 4) μg kg^−1^	(5.1 ± 2.3) μg kg^−1^	(23 ± 6) μg kg^−1^
PET-O-V	(202 ± 8) mg kg^−1^	(10 ± 1) μg kg^−1^	(1.4 ± 0.6) μg kg^−1^	(11 ± 2) μg kg^−1^
PET-T-V	(261 ± 3) mg kg^−1^	(20 ± 3) μg kg^−1^	(1.6 ± 0.8) μg kg^−1^	(21 ± 4) μg kg^−1^
RPET-T	(265 ± 1) mg kg^−1^	(23 ± 3) μg kg^−1^	(4.6 ± 1.9) μg kg^−1^	(27 ± 5) μg kg^−1^

**Table 3:** Details on analyzed plastic samples.

Sample Label	Polymer Type	Color	Origin
PET-B-E	PET	Blue	Environmental Plastic
PET-O-E	PET	Orange	Environmental Plastic
PET-T-E	PET	Transparent	Environmental Plastic
PET-B-V	PET	Blue	Virgin Plastic
PET-O-V	PET	Orange	Virgin Plastic
PET-T-V	PET	Transparent	Virgin Plastic
RPET-T-V	Recycled PET	Transparent	Virgin Plastic

## Data Availability

The dataset is available on request from the authors.

## Supplementary Materials

The following supporting information can be downloaded at https://www.mdpi.com/article/10.3390/molecules29122870/s1, Table S1: Summary of the experimental conditions and chromatographic features; Table S2: ICP-MS working parameters; Figure S1: Estimation of resolution and analysis time; Figure S2: Experimental versus fitted data resulting from multivariate regression; Figure S3: ATR-IR spectra of PET samples; Figure S4: AGREE scores for selected existing analytical methods; Figure S5: Pictures of the environmental plastic samples analyzed in this work.
