# Supplementary material for: Optimizing Antimony Speciation Analysis via Frontal Chromatography–ICP-MS to Explore the Release of PET Additives
Source: Molecules. 2024 Jun 16;29(12):2870. doi: 10.3390/molecules29122870 (PMC11207106; doi:10.3390/molecules29122870)

Supplementary Materials

Article

Optimizing Antimony Speciation Analysis via Frontal Chromatography – ICP-MS to Explore the Release of PET Additives

Alejandro R. López [1,2], Gilberto Binda [2,3], Gianluca Roncoroni [2], Sandro Recchia [2], Damiano Monticelli [2,*] and Davide Spanu [2,*]

[1] University School for Advanced Studies IUSS Pavia, 27100 Pavia, Italy; alejandro.ruiz@iusspavia.it
[2] Department of Science and High Technology, University of Insubria, Via Valleggio 11, 22100 Como, Italy; sandro.recchia@uninsubria.it (S.R.); groncoroni1@uninsubria.it (G.R.)
[3] Norwegian Institute for Water Research (NIVA), Økernveien 94, 0579 Oslo, Norway; gilberto.binda@niva.no
* Correspondence: davide.spanu@uninsubria.it (D.S.); damiano.monticelli@uninsubria.it (D.M.); Tel.: +39-0312386428 (D.S.); Tel: +39-0312386427 (D.M.)

Table S1. Summary of the experimental conditions explored during DoE analysis and corresponding resolution and overall analysis time.

Condition n°	Column	HNO_3 concentration (M)	Sample flow rate (mL min^{-1})	Resolution	Analysis time (s)
1	C1 (2×25 mm)	0.5	1.2	0.83	89
2	C1 (2×25 mm)	0.5	1.45	0.77	78
3	C1 (2×25 mm)	0.5	1.7	0.60	71
4	C1 (2×25 mm)	0.25	1.2	1.35	135
5	C1 (2×25 mm)	0.25	1.45	0.98	132
6	C1 (2×25 mm)	0.25	1.7	1.09	108
7	C1 (2×25 mm)	0.75	1.2	0.54	78
8	C1 (2×25 mm)	0.75	1.45	0.56	65
9	C1 (2×25 mm)	0.75	1.7	0.61	54
10	C2 (3×25 mm)	0.5	1.2	1.78	141
11	C2 (3×25 mm)	0.5	1.45	1.18	136
12	C2 (3×25 mm)	0.5	1.7	1.36	111
13	C2 (3×25 mm)	0.25	1.2	2.11	249
14	C2 (3×25 mm)	0.25	1.45	2.55	192
15	C2 (3×25 mm)	0.25	1.7	1.47	237
16	C2 (3×25 mm)	0.75	1.2	0.94	134
17	C2 (3×25 mm)	0.75	1.45	0.94	108
18	C2 (3×25 mm)	0.75	1.7	0.81	96
19	C3 (2×50 mm)	0.5	1.2	1.58	169
20	C3 (2×50 mm)	0.5	1.45	1.08	160
21	C3 (2×50 mm)	0.5	1.7	0.90	143
22	C3 (2×50 mm)	0.25	1.2	2.63	269
23	C3 (2×50 mm)	0.25	1.45	2.24	237
24	C3 (2×50 mm)	0.25	1.7	1.31	247
25	C3 (2×50 mm)	0.75	1.2	0.97	144
26	C3 (2×50 mm)	0.75	1.45	0.94	116
27	C3 (2×50 mm)	0.75	1.7	0.74	110
28	C4 (3×50 mm)	0.5	1.2	2.10	268
29	C4 (3×50 mm)	0.5	1.45	1.76	239
30	C4 (3×50 mm)	0.5	1.7	1.43	225
31	C4 (3×50 mm)	0.25	1.2	2.41	547
32	C4 (3×50 mm)	0.25	1.45	1.85	511
33	C4 (3×50 mm)	0.25	1.7	2.11	417
34	C4 (3×50 mm)	0.75	1.2	1.31	233
35	C4 (3×50 mm)	0.75	1.45	1.26	201
36	C4 (3×50 mm)	0.75	1.7	1.20	178

Table S2. ICP-MS working parameters.

Parameter	Value
RF Power	1550 W
Nebulizer gas flow	1.06 L/min
Auxiliary gas flow	0.80 L/min
Cooling gas flow	13.99 L/min
Dwell time	300 ms

Figure S1. Illustration of analytical estimation for resolution and analysis time. a) Frontal chromatogram depicting a Sb(III)-Sb(V) mixture solution, and b) derivative function utilized for simulating an elution chromatogram. Each peak is modeled with a Gaussian function (refer to equation in Figure b). Resolution and analysis time are quantified based on the equations provided in the Figure, adhering to the standard definitions for these parameters in elution chromatography.

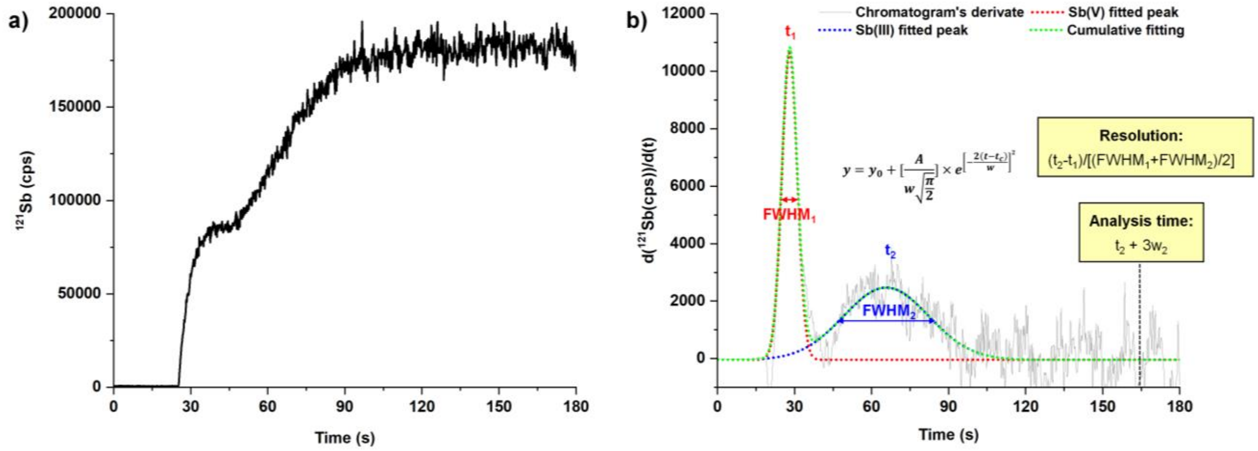

Figure S2. Scatter plots depicting Experimental versus Fitted resolution and analysis time data resulting from multivariate regression. Each data point is labeled with a number corresponding to the specific experimental condition (refer to Table S1 for details).

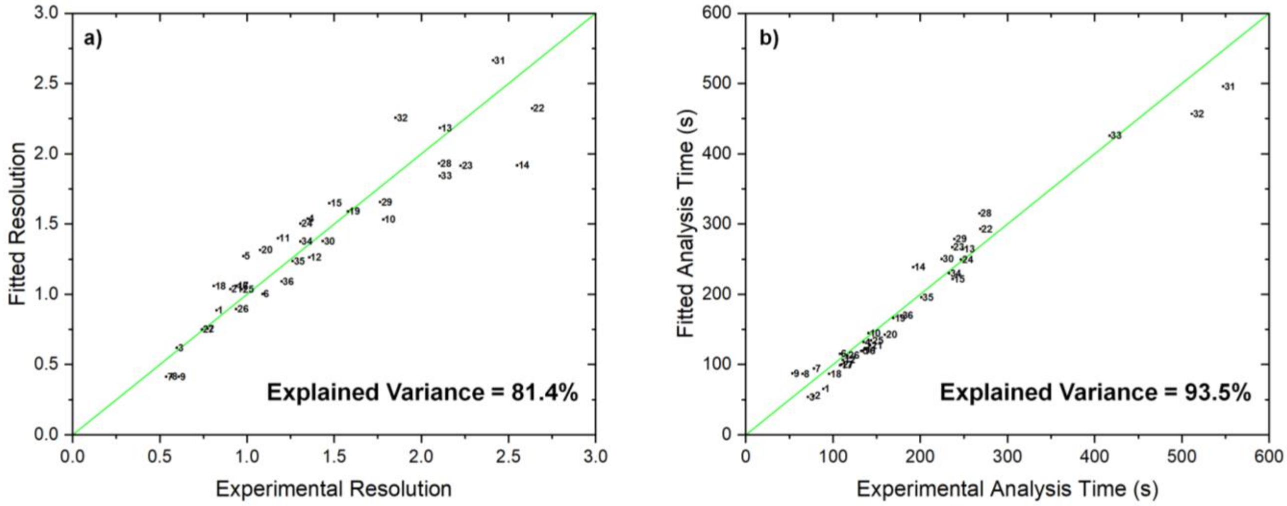

Figure S3. ATR-IR spectra recorded for all the analyzed samples.

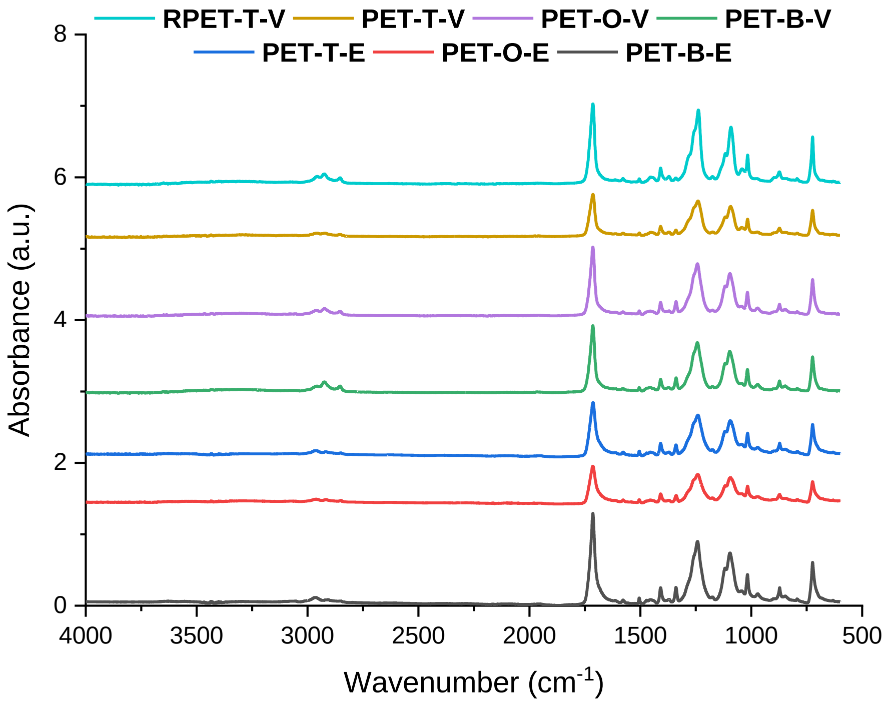

Figure S4. Results of AGREE analysis for two selected existing methods (see Ref. [28] for Figure a, and Ref. [54] for Figure b).

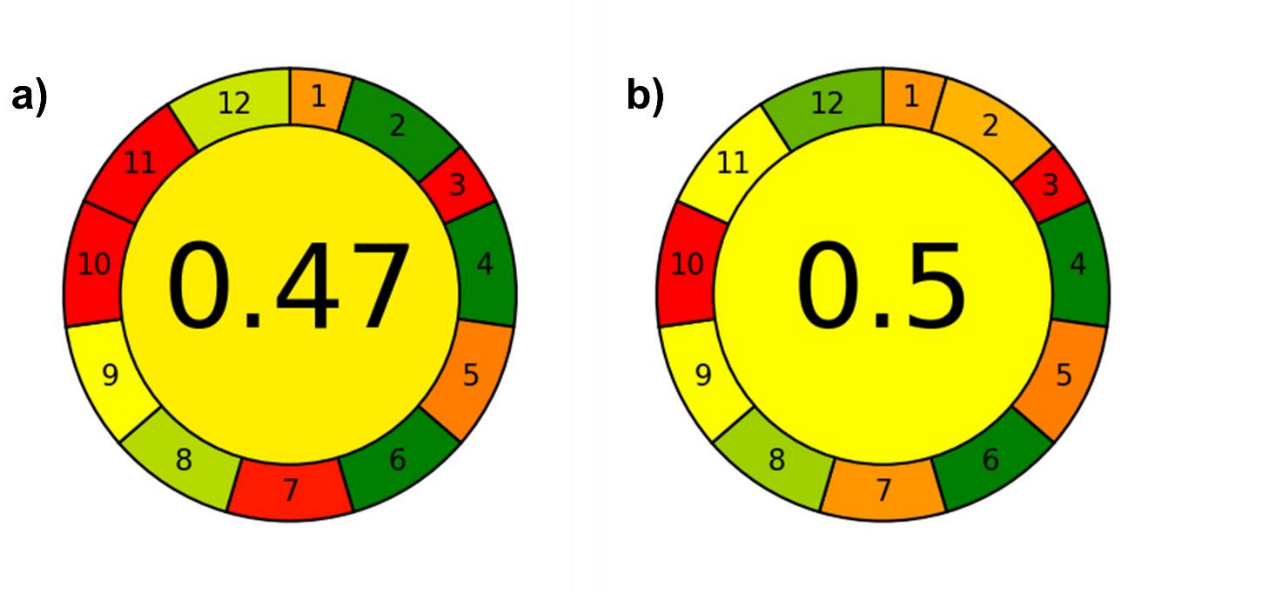

Figure S5. Pictures of the environmental plastic samples analyzed in this work.

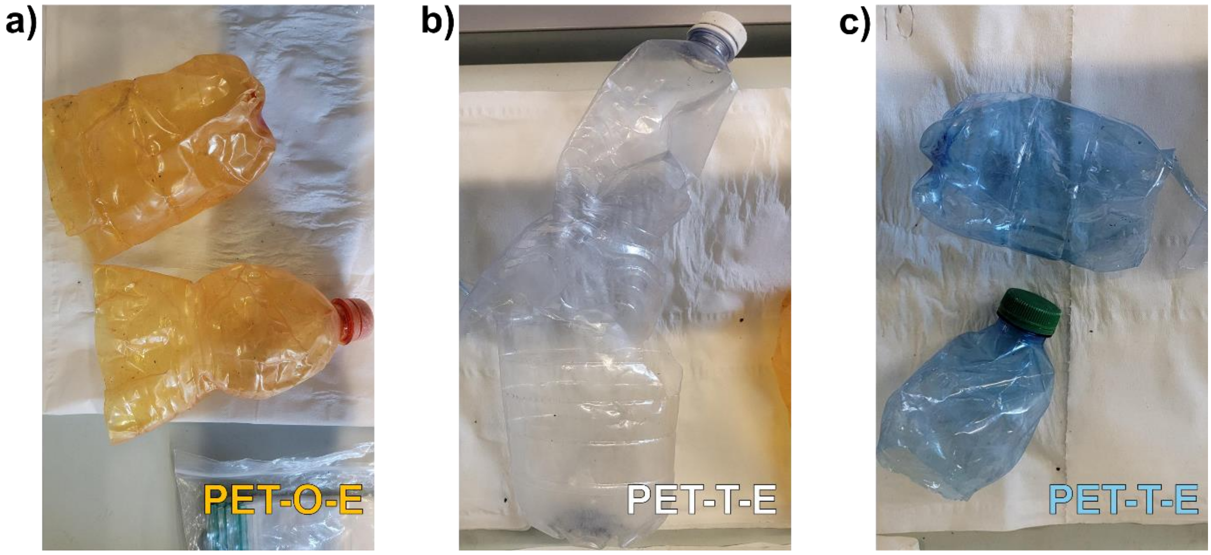

Supplement: Supplementary file 1 [file molecules-29-02870-s001.zip › molecules-3047043-supplementary.pdf]
